# Hydroxysafflor yellow A induced ferroptosis of Osteosarcoma cancer cells by HIF-1α/HK2 and SLC7A11 pathway

**DOI:** 10.32604/or.2023.042604

**Published:** 2024-04-23

**Authors:** YIWEN ZHU, LIU YANG, YING YU, YING XIONG, PING XIAO, XIAO FU, XIN LUO

**Affiliations:** 1 Department of Rehabilitation Medicine, The Second Affiliated Hospital of Nanchang University, Nanchang, 330006, China; 2 Department of General Practice, The Second Affiliated Hospital of Nanchang University, Nanchang, 330006, China

**Keywords:** Hydroxysafflor yellow A, Osteosarcoma, HIF-1α, FPT

## Abstract

Osteosarcoma is a very serious primary bone cancer with a high death rate and a dismal prognosis. Since there is no permanent therapy for this condition, it is necessary to develop a cure. Therefore, this investigation was carried out to assess the impacts and biological functions of hydroxysafflor yellow A (HYSA) in osteosarcoma cell lines (MG63). In this investigational study, MG63 cells were utilized. Microarray experiments, quantitative polymerase chain reaction (qPCR), immunofluorescent staining, extracellular acidification rate (ECAR), oxygen consumption rate (OCR), glucose consumption, lactate production, and ATP levels, proliferation assay, 5-Ethynyl-2′-deoxyuridine (EDU) staining, and Western blot were performed. In MG63 cells, HYSA lowered cell proliferation and metastasis rates, suppressed EDU cell number, and enhanced caspase-3/9 activity levels. HYSA reduced the Warburg effect and induced ferroptosis (FPT) in MG63 cells. Inhibiting ferroptosis diminished HYSA’s anti-cancer activities in MG63 cells. The stimulation of the HIF-1α/SLC7A11 pathway decreased HYSA’s anti-cancer activities in MG63 cells. HIF-1α is one target spot for HYSA in a model of osteosarcoma cancer (OC). HYSA altered HIF-1α’s thermophoretic activity; following binding with HYSA, HIF-1α’s melting point increased from ~55°C to ~60°C. HYSA significantly enhanced the thermal stability of exogenous WT HIF-1α while not affecting Mut HIF-1α, suggesting that ARG-311, GLY-312, GLN-347, and GLN-387 may be involved in the interaction between HIF-1α and HYSA. Conclusively, our study revealed that HYSA induced FPT and reduced the Warburg effect of OC through mitochondrial damage by HIF-1α/HK2/SLC7A11 pathway. HYSA is a possible therapeutic option for OC or other cancers.

## Introduction

Osteosarcoma is a malignant tumor originating from bone tissue. It is composed of tumorous spindle stromal cells and osteoid [1]. It often invades the metaphysis of long bones, particularly the distal femur and proximal tibia [2]. It is prone to hematogenous lung metastasis and is prone to occur in adolescents [2]. Osteosarcoma can have distant metastasis at an early stage, with high mortality and poor prognosis [2,3]. Osteosarcoma aetiology is complicated. While the five-year survival rate has increased as medical technology has advanced, no significant gain has been achieved [4]. Recent research has associated the disruption of different intracellular signal pathways with the formation and progression of osteosarcoma [5,6]. It is critical to investigate the signal pathways involved in the incidence, progression, and therapy of osteosarcoma [5].

FPT, a recently found kind of cellular death, has been associated with the formation and progression of osteosarcoma [7]. Its mechanism involves iron, lipid metabolism, oxidative stress, glutathione (GSH), coenzyme Q10 production, and other biological processes, and it regulates osteoclasts and osteosarcoma cells [8].

It is critical to investigate the pathophysiology of these two illnesses from a cellular standpoint. Hypoxia and its associated factor HIF-1α play a significant role in regulating the transcription of various genes that are targeted by tumors. It has a profound effect on the energy metabolism, proliferation, and apoptosis of tumour cells [9–11]. Therefore, it is essential to successfully counteract the function that hypoxia plays in tumour cell death [12]. Safflower injection is processed from safflower, a plant of the composite family, through water extraction, alcohol precipitation, concentration, water precipitation, sterilization, etc. [13]. Modern pharmacology has proved that it has pharmacological effects such as activating blood circulation and removing stasis, antioxidation, anti-inflammatory, and lowering blood pressure and blood lipids [14]. Hydroxysafflower yellow A (HYSA) was isolated from safflower for the first time, which belongs to flavonoids and is the main water-soluble component in safflower [15]. Pharmacological and clinical studies have shown that HSYA has anti-inflammatory, antioxidant, platelet aggregation-inhibitory, and other effects. It is applied to treating ischemic heart disease, preventing hypoxia injury, and protecting against chronic liver fibrosis [16]. It was discovered that HSYA suppressed the aberrant growth of human umbilical vein endothelial cells (HUVEC) created from the supernatant of colorectal cancer cells by monitoring the proliferation and death of HUVEC [17]. HSYA regulates the expression of downstream genes by inhibiting the ERK1/2 signal transduction pathway, thus inducing apoptosis in hepatic stellate cells. HSYA can reduce tumor cell proliferation and promote cell apoptosis, thereby decreasing the development of human gastric cancer cells [18]. By decreasing tumor microvessel density, HSYA may impede tumor development [19]. This study aimed to investigate the involvement of HIF-1α, HK2, and the SLC7A11 pathway. Hypoxia-inducible factor-1 (HIF-1) has been previously acknowledged as a significant target for cancer therapeutics [20]. HK2 has been identified as a potential regulator of osteoarthritis through both glycolytic and non-glycolytic pathways [21]. The expression of SLC7A11 is observed in various tissues and it serves multiple functional roles in the pathophysiology of different diseases, such as cancer. Its regulatory functions encompass redox homeostasis, metabolic flexibility/nutrient dependency, immune system function, and ferroptosis [22]. But there is no previous research on ferroptosis in Osteosarcoma cancer. Therefore, this research was to examine the impacts and biological processes of HYSA in osteosarcoma.

## Materials and Methods

### Study area

The present study was carried out in the Second Affiliated Hospital of Nanchang University, Nanchang, Jiangxi, 330006, China from January–April 2022.

### Cell culture

In this investigation, osteosarcoma cell lines (MG63) were obtained from (ZQXZBIO, Shanghai, China) and grown in accordance with American Type Culture Collection (ATCC) approaches at 37°C in a 5% CO_2_ environment. Various levels of HYSA (0, 2.5, 5, and 10 µM) were applied to MG63 cells and incubated for 48 h.

### qPCR

Whole RNAs were extracted using the RNA isolator total RNA separation reagent, and PrimeScipt RT Master Mix (Takara, China) was employed to create cDNA. The qPCR was conducted utilising the ABI Prism 7500 sequencer reader and the Prime-ScriptTM RT identification kit. The levels of mRNA expression in the samples were computed and represented as 2-DDCt.

### Immunofluorescent staining

The cells were maintained for 24 h with 5% O_2_, 5% CO_2_, and 90% N_2_. It was then fixed for 15 min in 4% paraformaldehyde before being treated for 15 mins in 0.15% Triton X-100 at ambient temperature. After 1 h of inhibition with 5% BSA, cells were treated throughout the night at 4°C with HIF-1 (1:500, ab1, Abcam) and SLC7A11 (1:500, ab275411, Cell Signalling Technology, Danvers, Massachusetts, USA). Cells were treated for 2 h at ambient temperature with goat anti-rabbit IgG-cFL 488 or anti-rabbit IgG-cFL 555 antibody (1:100), labelled with DAPI for 15 min, and finally rinsed with PBS for 15 min. Cell pictures were captured utilising Carl Zeiss Axioplan 2 fluorescent microscope (Carl Zeiss AG, Oberkochen, Germany).

### Assessment of ECAR, OCR, glucose uptake, lactate, and ATP levels

The following parameters were assessed in this investigation: ECAR, OCR, glucose absorption, lactate generation, and ATP level were performed as per the descriptions of Pu et al. [23]. ECAR and OCR were assessed by utilizing the Seahorse XFe96 analyzer (Seahorse Bioscience, Agilent). The glucose level was assessed by utilising the glucose assessment kit (Sigma, St-Louis, MO, USA). The Lactate Colorimetric/Fluorometric Assay Kit (BioVision, Mountain View, CA, USA) was used to assess the lactate levels. The ATP Determination Kit (Thermo Fisher Scientific, USA) was utilized to analyze the ATP level.

### Assessment of LDH, Fe^2+^, ROS and GSH levels

LDH, Fe^2+^, and GSH levels have been assessed using the techniques outlined by Wang et al. [24]. LDH, MDA, Fe^2+^, ROS, and GSH levels in cells were assessed by utilising a microplate analyzer at absorption wavelengths of 490, 532, 593, 490, and 412 nm.

### Mitochondrial damage

In this study, mitochondrial damage was carried out by the method of Guo et al. [25].

### Proliferation assessment and 5-ethynyl-2′-deoxyuridine (EDU) staining

In this study, around 2 × 10^3^ cells were plated in each well of the 96-well plate. After growing for the following time frames (0, 1, 2, 3, and 4 days), the cells were measured by the CellTiter-GloR Luminescent Cell Viability assessment kit (Promega, Madison, WI, USA) as per the kit instructions. Each well was treated with 10 mM EdU before being fixed in 4% formaldehyde for 30 min. After rising, EdU was identified by the Click-iTR EdU Kit (Thermo Fisher Scientific, China), and pictures were taken with an Olympus fluorescence microscope.

### Mitochondrial permeability transition (MPT) assessment

The MPT was assessed by raising intracellular calcium by utilising 0.5 mM ionomycin calcium salt (Molecular Probes, Life Technologies, Carlsbad, USA) following the method of Treulen et al. [26].

### Transwell assay

The cells were introduced in the top chamber containing Dulbecco's Modified Eagle Medium (DMEM), and in the bottom chamber, containing 600 μL media with 10% of fetal bovine serum (FBS). The cells were cultured in standard circumstances for 24 h and fixed in methanol for 10 min then treated with 0.1% w/v crystal violet for 30 min and then rinsed with water. Finally, the cells were photographed by a fluorescent microscope (Olympus).

### Western blot

After inhabiting with 5% BSA in TBS, the membranes were treated with the primary antibodies: GPX4 (1:1000), HIF-1α (1:500), SLC7A11 (1:500), and β-Actin (1:5000). Then it was incubated with peroxidase-associated with secondary antibodies. The ECL system received the signals, which were pictured by the ChemiDoc XRS system by Image Lab software (Bio-rad).

### Anti-cancer effects of HYSA in a model of MG63 cells by using FPT inhibitor

In this study, the anti-cancer effects of HYSA in the MG63 cells by using an FPT inhibitor were assessed by the method of Yang et al. [27].

### Microscale thermophoresis (MST), thermal shift assay (TSA) and cellular thermal shift assay (CETSA)

WT HIF-1α protein (0.10 mg/mL) was used with or without 50 μM HYSA in PBS. Mut HIF-1 plasmids ARG-311, GLY-312, GLN-347, and GLN-387 were introduced into HEK 293T cells utilising LTX and PLUS reagents (Invitrogen) then the cells were cultivated for a further 2 h with 100 mol/L of HYSA A after 8 h, whereas control cells underwent incubation in an equal quantity of PBS. The cells were grown in PBS (containing 1 mmol/L PMSF) and heated for 3 min using a temperature gradient ranging from 37°C to 67°C. Western blotting was performed using 20 L of supernatant. CETSA and thermal resistance were assessed using GraphPad Prism (GraphPad, San Diego, California, USA).

### Molecular docking assay

Molecular docking was employed to understand the active sites and forces responsible for the interaction between drug and protein. Docking is frequently utilised to indicate the protein inhibitors’ binding mechanisms. Docking was carried out by utilising the PyRx Virtual Screening tool with AutoDock Vina [28].

### Statistical analyses

In the study, GraphPad Prism 6 was employed. Student’s *t*-test and one-way analysis of variance (ANOVA) were employed to assess the comparative data, which were then evaluated by Tukey’s post hoc test. The Kaplan-Meier approach and the log-rank assessment for survival curves were utilised for survival assessment. The threshold level was fixed at *p* < 0.05.

## Results

### HYSA reduced cell growth of OC cells

**Figure 1:**
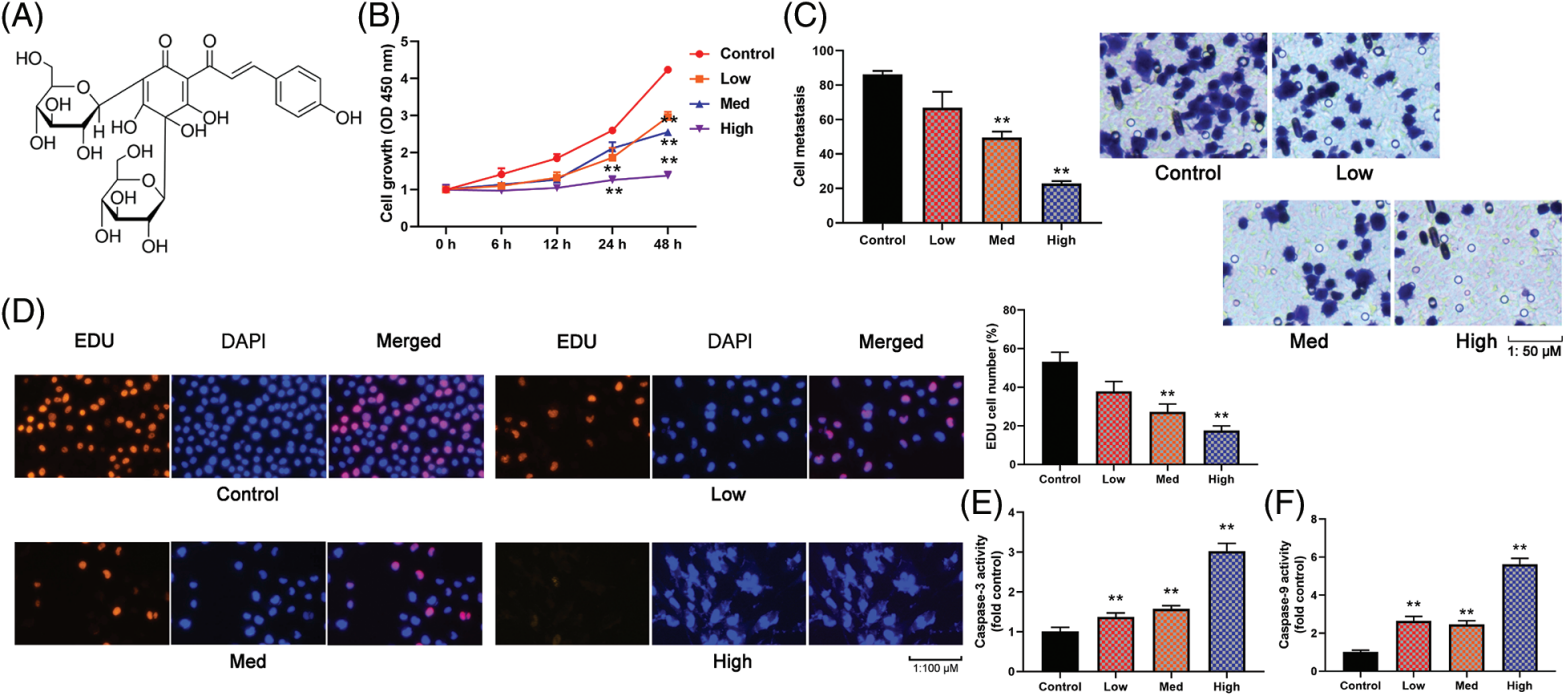
HYSA reduced cell growth of OC cells. Compound structure of HYSA (A), cell growth (B), cell metastasis rate (C), EDU cell number (D), caspase-3/9 activity levels (E and F) of OC cells. Control, Low, Med and High: 0, 2.5, 5, and 10 μM of HYSA for 24 h. **p < 0.01 compared with the control group.

The study explored the possible role of HYSA in a model of OC cells (MG63 cells). Fig. 1A expresses the Compound structure of HYSA. HYSA suppressed EDU cell quantity and lowered cell development and metastatic rates in MG63 cells. Fig. 1B illustrates that the MG63 cells expressed the highest growth rate in 48 h. Fig. 1C depicts the cell metastasis rate. The highest value of HYSA inhibited the development of MG63 cells (Fig. 1D). In the meantime, HYSA increased caspase-3/9 expression in MG63 cells. (Figs. 1E and 1F). These findings demonstrated that HYSA inhibited MG63 cell proliferation.

### HYSA reduced the Warburg effect in MG63

**Figure 2:**
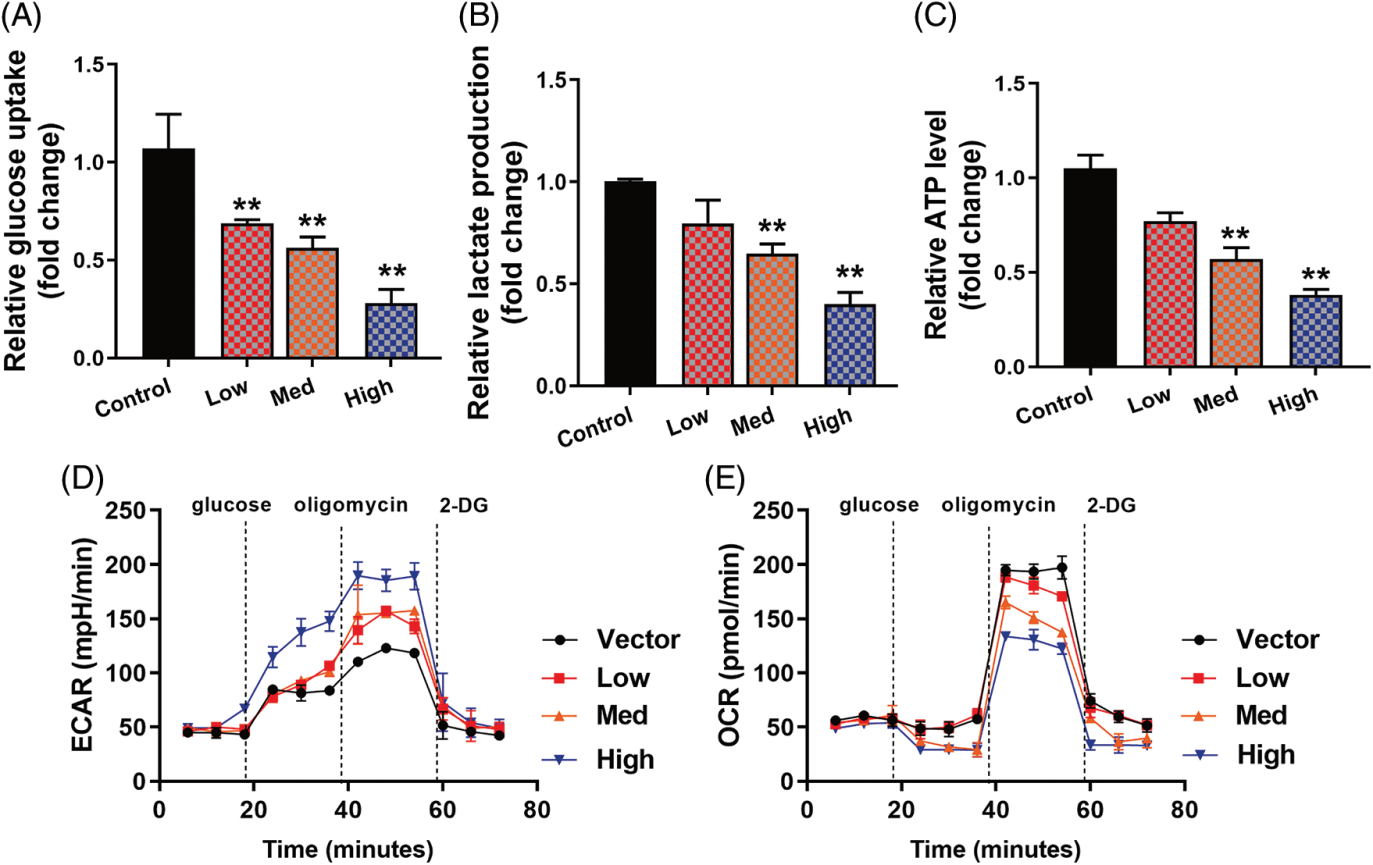
HYSA reduced the Warburg effect of OC cells. Glucose consumption (A), lactate production (B), ATP quantity (C), lactate-induced acidification of the medium surrounding cells (D), mitochondrial respiratory capacity (E). Control, Low, Med and High: 0, 2.5, 5, and 10 μM of HYSA for 24 h. **p < 0.01 compared with the control group.

In this investigation, the highest concentration of HYSA decreased the amount of glucose uptake (Fig. 2A), while the lowest concentration of HYSA increased the level of glucose uptake. In the case of lactate production (Fig. 2B), the highest concentration of the HYSA decreased the amount of relative lactate production, while the lowest concentration of the HYSA increased the level of relative lactate production. The highest concentration of the HYSA decreased the relative ATP level, and a lower concentration of the HYSA produced a higher ATP level in MG63 cells (Fig. 2C). HYSA also boosted OCR and decreased the extracellular acidification rate (ECAR) of the surrounding media caused by lactate (Figs. 2D and 2E). Taken together, it suggested that HYSA reduced the Warburg effect of MG63 cells.

### HYSA-induced FPT of MG63 cells

**Figure 3:**
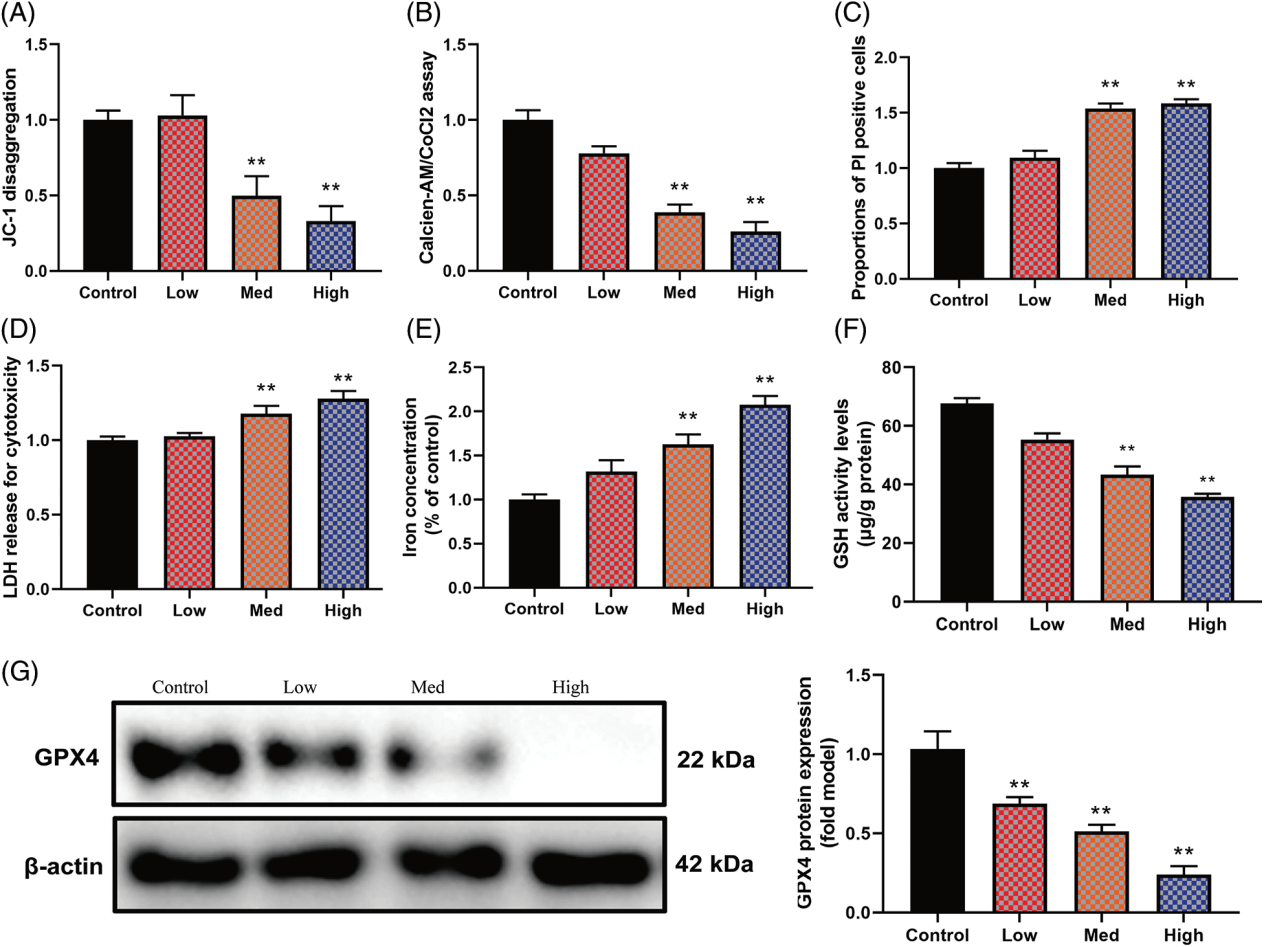
HYSA-induced ferroptosis of OC cells. Mitochondrial damage (A), MPT (B), PI-positive cells (C), LDH activity level (D), lron concentration level (E), GSH and GPX4 protein expressions (F and G). Control, Low, Med and High: 0, 2.5, 5, and 10 μM of HYSA for 24 h. **p < 0.01 compared with the control group.

In this investigation, the impact of HYSA on MG63 cells’ FPT was assessed in Fig. 3. In MG63 cells, the highest concentration of HYSA expressed low mitochondrial damage (JC-1 disaggregation). At the same time, a lower concentration of HYSA expressed high mitochondrial damage (Fig. 3A); the highest concentration of HYSA expressed a lower Mitochondrial Permeability transition; and a lower concentration of HYSA expressed a higher Mitochondrial Permeability Transition (calcein AM/CoCl2 test) (Fig. 3B). At the same time, the highest concentration of HYSA expressed a high proportion of PI-positive cells (Fig. 3C), high LDH release for cytotoxicity (Fig. 3D), and a higher level of concentration (Fig. 3E). The highest concentration of HYSA expressed a lower amount of GSH and GPX4 proteins, while the lower concentration of HYSA expressed a high level of GSH and GPX4 proteins (Figs. 3F and 3G).

### FPT reduced the anti-cancer effects of HYSA in a model of MG63 cells

**Figure 4:**
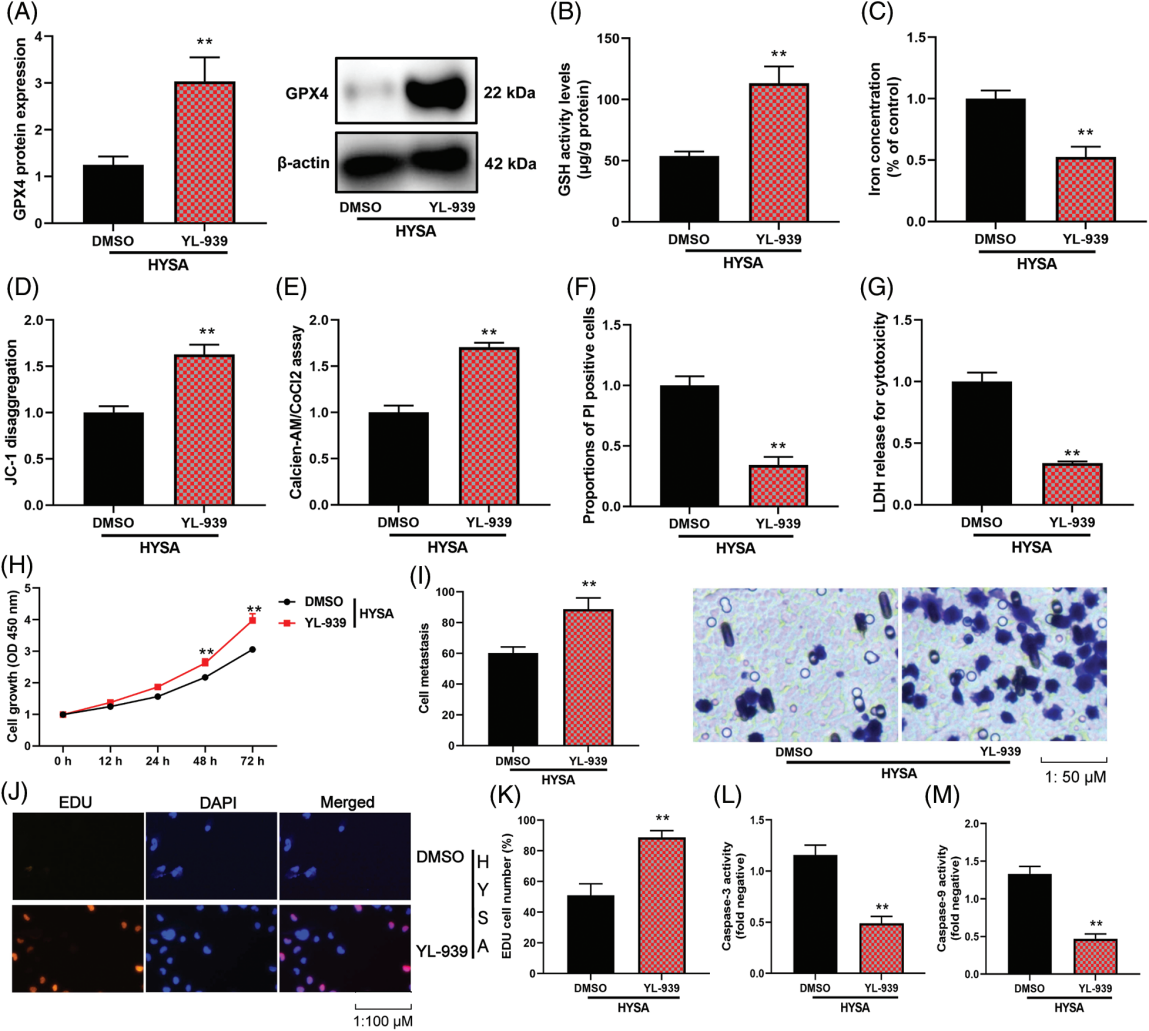
The inhibition of ferroptosis reduced the anti-cancer effects of HYSA in a model of OC. GPX4 protein expressions (A), GSH (B), lron concentration level (C), Mitochondrial damage (D), MPT (E), PI-positive cells (F), LDH activity level (G), cell growth (H), cell metastasis rate (I), EDU cell number (J and K), caspase-3/9 activity levels (L and M) of OC cells. HYSA, 5 μM of HYSA for 24 h; **p < 0.01 compared with the DMSO group.

This study shows that FPT is functionally involved in HYSA in the treatment of OC. In this investigation, YL-939 (5 µM) was used as an FPT inhibitor, and it showed a high level of GPX4 protein expression (Fig. 4A), GSH activity level, JC-1 disaggregation (mitochondrial damage), Mitochondrial Permeability Transition (calcein AM/CoCl2 test), but reduced iron concentration level, the proportion of PI-positive cells, and LDH release for cytotoxicity (Figs. 4A–4G). Fig. 4H illustrates that the MG63 cells expressed the highest growth rate in 72 h. Fig. 4C depicts that YL-939 expresses a high cell metastasis rate. The FPT inhibitor increased the number of EDU cells, enhanced the pace of cell growth and metastasis (Fig. 4J), and decreased the levels of caspase-3/9 activity by administering HYSA to OC cells (Fig. 4M). These data showed that HYSA reduced the cell growth of OC cells through the promotion of FPT.

### HYSA suppressed HIF-1α/SLC7A11 pathway

**Figure 5:**
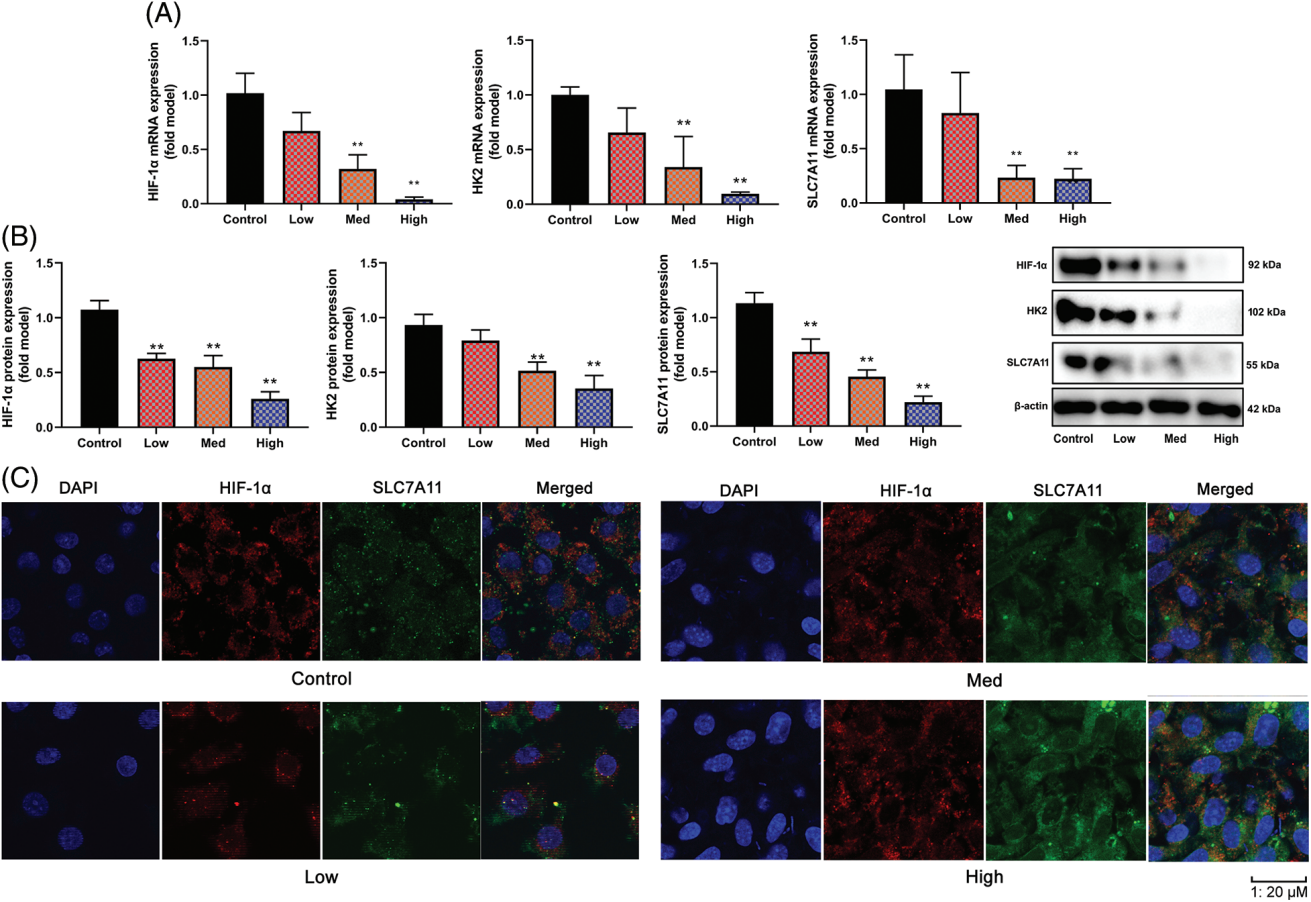
HYSA suppressed HIF-1α/SLC7A11 pathway. HIF-1α/HK-2/SLC7A11 mRNA expression (A), HIF-1α/HK-2/SLC7A11 protein expression (B), HIF-1α/ SLC7A11 expression (C, Immunofluorescence). Control, Low, Med and High: 0, 2.5, 5, and 10 μM of HYSA for 24 h. **p < 0.01 compared with the control group.

In this investigation, the Warburg effect and FPT of OC were examined together with the impacts and mechanisms of HYSA. In this experimental study, it was observed that in OC cells, the expression of HIF-1α, HK2, and SLC7A11 mRNA and protein was reduced when high concentrations of HYSA were administered. Conversely, low concentrations of HYSA resulted in elevated levels of expression of HIF-1α, HK2, and SLC7A11 mRNA and protein. These effects were found to be dependent on the NAD+/Sirt1 cascades, which play a role in mitigating oxidative stress. This is illustrated in Figs. 5A and 5B. Then, HYSA also reduced nuclear localization of HIF-1α/SLC7A11 expression in OC cells (Fig. 5C). Taken together, our observations suggest that HYSA suppresses the HIF-1α/SLC7A11 pathway to regulate the Warburg effect and FPT of OC.

### The activation of the HIF-1α/SLC7A11 pathway reduced the anti-cancer properties of HYSA in a model of OC

**Figure 6:**
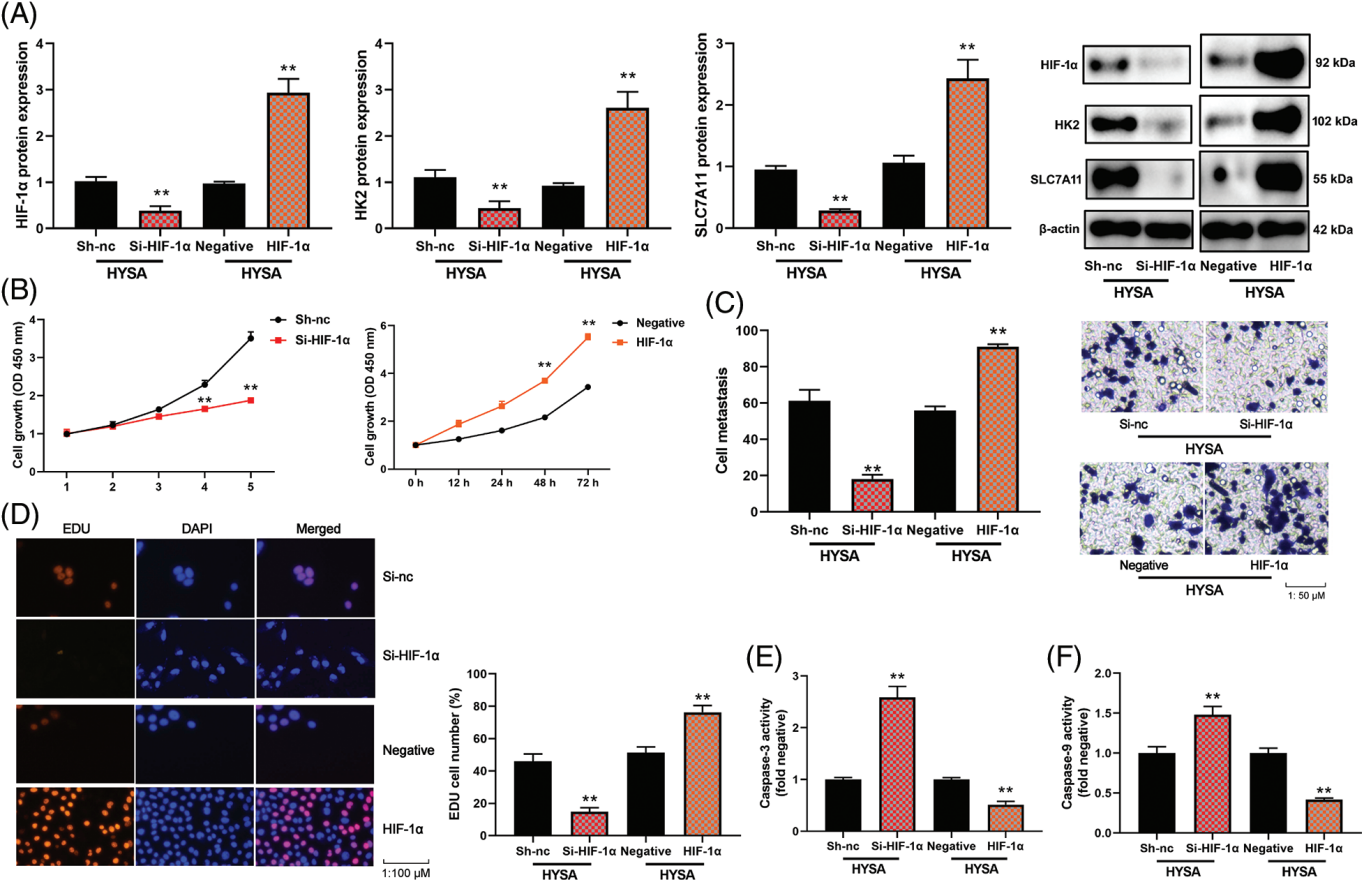
The activation of the HIF-1α/SLC7A11 pathway reduced the anti-cancer effects of HYSA in a model of OC. HIF-1α/HK-2/SLC7A11 protein expression (A), cell growth (B), cell metastasis rate (C), EDU cell number (D), caspase-3/9 activity levels (E and F) of OC cells. HYSA, 5 μM of HYSA for 24 h; **p < 0.01 compared with the negative or si-nc group.

**Figure 7:**
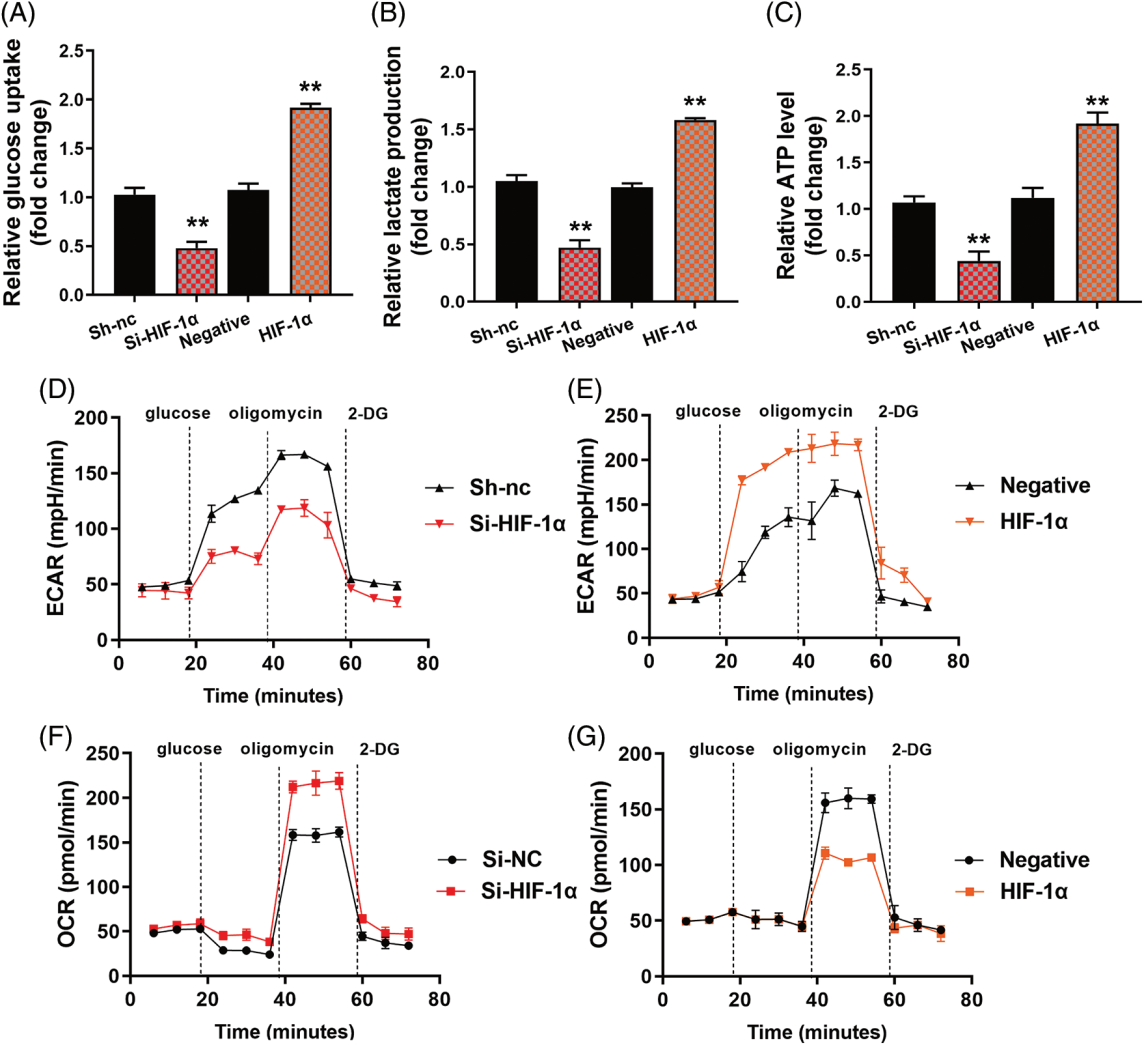
The activation of the HIF-1α/SLC7A11 pathway reduced the anti-cancer effects of HYSA on the Warburg effect of OC. Glucose consumption (A), lactate production (B), ATP quantity (C), lactate-induced acidification of the medium surrounding cells (D and E), mitochondrial respiratory capacity (F and G) HYSA, 5 μM of HYSA for 24 h; **p < 0.01 compared with the negative or si-nc group.

The study evaluated the significance of HIF-1α in this function of HYSA in the model of OC. HIF-1α or si-HIF-1α plasmids regulated the expression of HIF-1α, HK2 and SLC7A11 mRNA expressions in OC cells by treatment with HYSA (Fig. 6A). Fig. 6B illustrates the low growth rate of the Si-HIF-1α plasmid. But HIF-1α expressed a high growth rate in the 72 h. Si-HIF-1α plasmid decreased cell proliferation and metastatic rate, suppressed EDU cell count (Fig. 6C), and elevated caspase-3/9 activity levels by treating OC cells with HYSA. HIF-1α plasmid promoted cell proliferation and metastatic rate, increased EDU cell count and decreased caspase-3/9 activity levels by treating OC cells with HYSA (Figs. 6E and 6F). Meanwhile, the si-HIF-1α plasmid reduced the Warburg effect of OC cells by HYSA. Fig. 7 shows that activation of the HIF-1α/SLC7A11 pathway reduced the anti-cancer effects of HYSA on the Warburg effect of OC. In this study, Si-HIF-1α expressed reduced relative glucose uptake, relative lactase production, and relative ATP level, while HIF-1α expressed a high level of relative glucose uptake, relative lactase production, and relative ATP level (Figs. 7A–7C). Figs. 7D and 7E express high ECAR levels by Sh-nc and HIF-1α at 40–60 min. Figs. 7F and 7G express a high oxygen consumption rate by the si-HIF-1α plasmid and is negative at 40–60 min.

**Figure 8:**
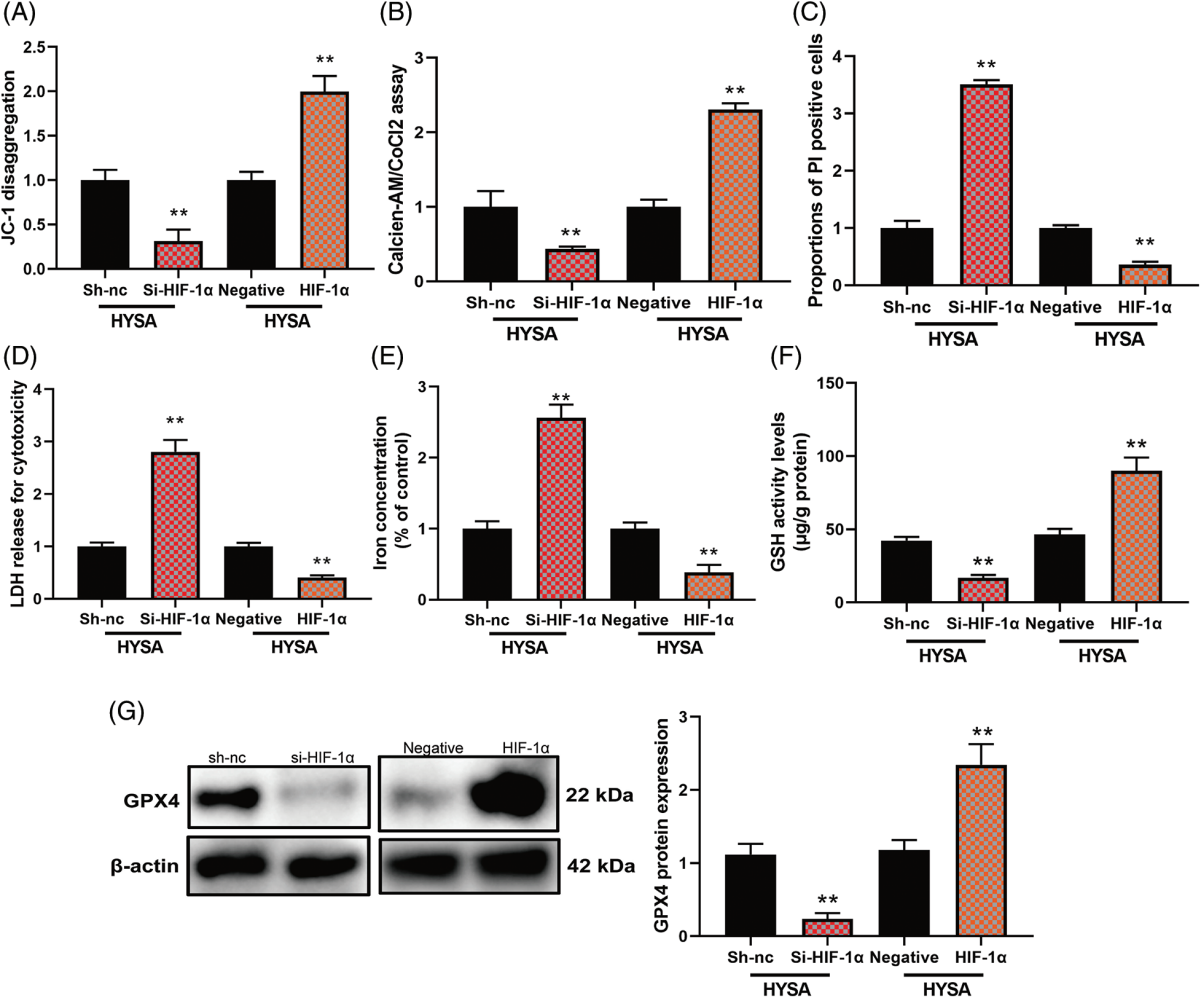
The activation of the HIF-1α/SLC7A11 pathway reduced the anti-cancer effects of HYSA on ferroptosis of OC. Mitochondrial damage (A), MPT (B), PI-positive cells (C), LDH activity level (D), lron concentration level (E), GSH and GPX4 protein expressions (F and G). Mitochondrial damage (A), MPT (B), PI-positive cells (C), LDH activity level (D), lron concentration level (E), GSH and GPX4 protein expressions (F and G). **p < 0.01 compared with the negative or si-nc group.

In this study, HIF-1α expressed high mitochondrial damage and mitochondrial Permeability transition (calcein AM/CoCl2 test) by HIF-1α but Si-HIF-1α reduced mitochondrial damage (Figs. 8A and 8B). At the same time, HIF-1α expressed a low level of proportion of PI-positive cells, LDH release for cytotoxicity, and iron concentration level, while Si-HIF-1α expressed a high level of proportion of PI-positive cells, LDH release for cytotoxicity, and iron concentration level. Figs. 8G–8F show higher GSH levels and GPX4 protein expression by HIF-1α. At the same time, Si-HIF-1α expressed lower levels of GSH and GPX4 protein expression in OC cells by HYSA (Fig. 8). These data showed that HYSA reduced the Warburg effect and promoted FPT of OC cells by HIF-1α/HK-2/SLC7A11 pathway.

### HIF-1α is one target spot for HYSA in a model of OC

**Figure 9:**
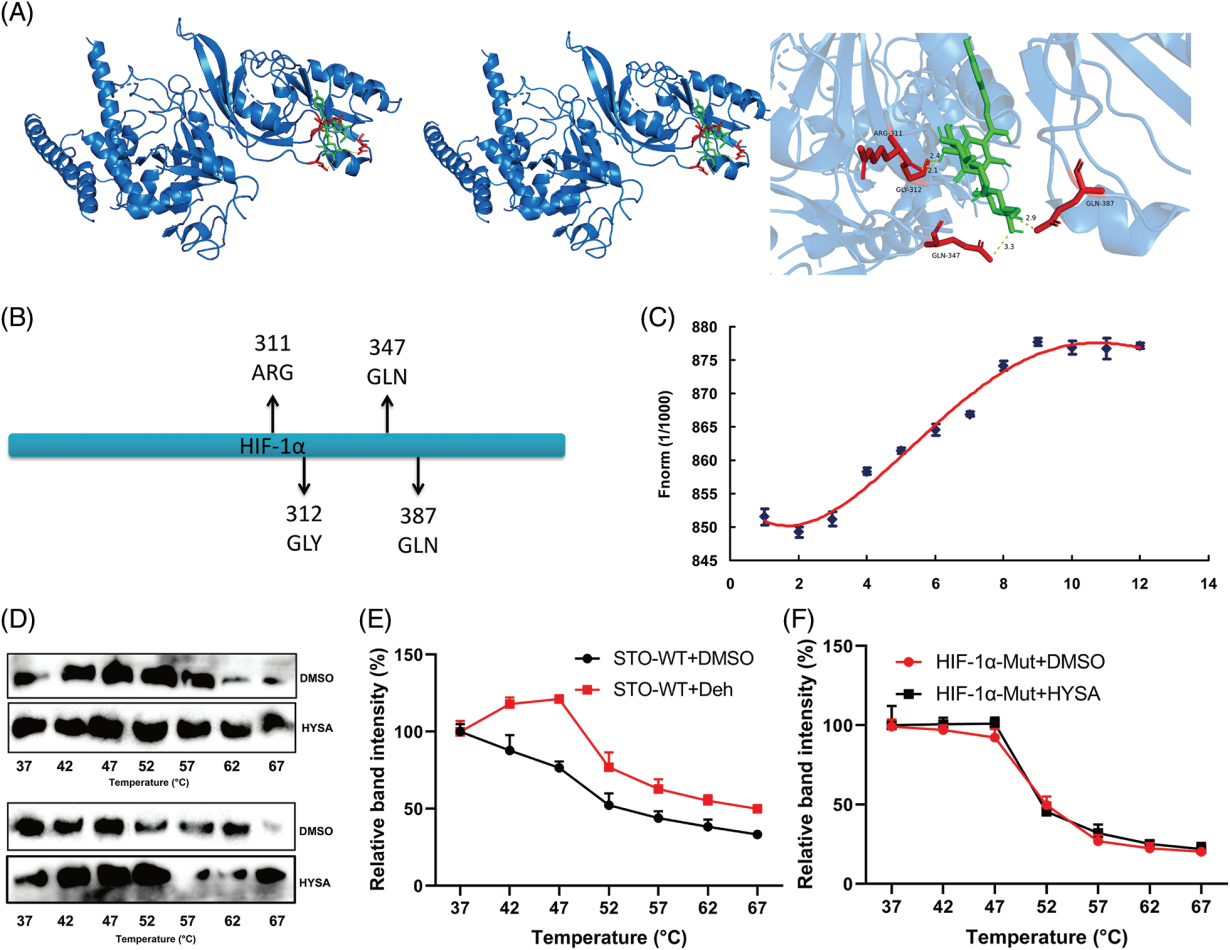
HIF-1α is one target spot for HYSA in a model of OC. The 3D image revealed that HYSA bonded to the binding pocket and formed with HIF-1α (A and B); HYSA incubated with HIF-1α (C, Microscale thermophoresis); TSA results in the presence or absence of HYSA (D); the thermal stability of WT HIF-1α and Mut HIF-1α (E and F).

The research investigated the significance of the HIF-1α signalling system in the impact of HYSA on the Warburg effect and FPT of OC. HYSA linkage HIF-1 was discovered using drug and protein linkage assessment, revealing that ARG-311, GLY-312, GLN-347, and GLN-387 may be accountable for the relationship between HIF-1α and HYSA (Figs. 9A and 9B). HYSA altered HIF-1α thermophoretic mobility; following binding with HYSA, HIF-1α melting point increased from ~55°C to ~60°C (Figs. 9C and 9D). CETSA experiments with HEK293T cells revealed that HYSA significantly increased the heat stability of exogenous WT HIF-1α (Figs. 9E and 9F). In this investigation, HIF-1α was identified as a potential target for the impacts of HYSA on the Warburg effect and FPT of OC.

## Discussion

Osteosarcoma is a highly prevalent bone tumor in people of all ages [29]. The traditional clinical treatment schemes mainly include preoperative chemotherapy, surgical limb salvage, and postoperative adjuvant chemotherapy [30]. However, in the last 20 years, the average survival rate of patients has remained stable at 65%–70% with no significant improvement [31]. It is highly sensitive to a variety of chemotherapy drugs, such as cisplatin, adriamycin, cyclophosphamide, etc., but it is easy to become resistant [32]. The key clinical characteristics of osteosarcoma are high tumor growth, ease of recurrence and metastasis, and the presence of abundant blood vessels at the site of occurrence [33]. Once the disease occurs, it can rapidly proliferate and transfer through cells, which is extremely invasive to the human body. About 35% of patients will face the risk of amputation [34]. With the continuous development of molecular biology and the continuous promotion of basic research, we have a preliminary understanding of many signal pathways and the regulatory mechanisms of osteosarcoma growth, proliferation, metastasis, invasion, and so on [35]. Numerous *in vivo* and *in vitro* experiments demonstrate the enormous potential of targeted medications, and new regulatory mechanisms and action targets have been identified [36]. In this study, HYSA decreased the rates of cellular growth and metastasis as well as the quantity of EDU cells in OC cells. Khojastehnezhad et al. [37] demonstrated that HYSA inhibits colorectal cancer cell proliferation and spread. Based on these findings, it has been hypothesised that HYSA inhibits the proliferation of OC cells and could be a potential drug. In recent years, the management of tumour energy metabolism has risen to the forefront of cancer prevention and therapeutic research [38,39]. The “Warburg effect” is a unique metabolic mode of tumor cells; autophagy is an important material metabolic mode and protective mechanism of cells; and autophagy defects promote tumor occurrence [40]. The data from this study showed that HYSA reduced the Warburg effect of OC cells. HYSA might be one potential drug for the Warburg effect of OC.

Previously, several investigations have demon-strateed that promoting cell FPT is a promising method for reducing cancer cell drug resistance. FPT is a novel, non-apoptosis-regulated mode of cell death character-ised by the aberrant deposition of iron-dependent lipid oxides, which has potential uses in the therapy of cancer, particularly interstitial tumors [41,42]. *In vivo* and *in vitro* experiments demonstrate that FPT has distinct physiological effects on pathological cellul-ar death related to degenerative diseases (such as Alzheimer’s, Huntington’s, and Parkinson’s), stroke, cerebral haemorrhage, traumatic brain damage, ischemia-reperfusion damage, mammalian kidney degeneration, and various types of tumours [43]. FPT is a new cell death mode characterised by iron depend-ence, active oxygen generation, and lipid peroxidation accumulation [44]. In this present study, HYSA induced FPT of OC cells. Chen et al. suggested that HYSA alleviated FPT in PC12 cells [37]. Based on these results, HYSA might be one potential drug for FPT for OC. Warburg effect’s new strategy for tumor treatment includes targeting the HIF-1α Signal pathway to regulate HIF-1α and its downstream effector molecular level, targeting oncogenes and tumor inhibitor genes to change the metabolic mode of cancer cells, directly interfering with aerobic glycolysis to block the energy supply of tumor cells, and targeting the acidic microenvironment to inhibit tumor invasion and metastasis [45,46]. Aerobic glycolysis is the basis of tumor cell proliferation and other biological functions. HK2 is the key enzyme in aerobic glycolysis and is overexpressed in tumors [47]. Inhibiting HK2 can inhibit tumor growth, regulate the Warburg effect, and improve tumor drug resistance [48]. FPT regulates the development and proliferation of tumour cells, which is critical in the formation and advancement of many tumours. FPT is strongly connected to SLC7A11 and GPX4 [49,50]. Cystine-glutamate reverse transporter is a crucial regulator of FPT in cells, and SLC7A11 is an essential part of the Cystine-glutamate system. Through this study, it has been found that HYSA suppresses the HIF-1α/SLC7A11 pathway. HIF-1α is one target spot for HYSA in the OC model. Analysis of drug and protein interactions revealed that HYSA interacts with HIF-1α, suggesting that ARG-311, GLY-312, GLN-347, and GLN-387 may be responsible for this interaction. Through this investigation, it was proven that the HIF-1α is one target spot for HYSA in the model of OC.

## Conclusion

In the end, our research provides direct confirm-ation that HYSA inhibits the warburg effect and FPT in OC via the HIF-1/SLC-A11 pathway. This research also revealed that HIS is a possible therapy for OC and other malignancies; however, more investigation about the particular mechanism has to be carried out in the future.

## Data Availability

The information used to justify this investigation is provided upon request.
